# Optimizing first-line TKI treatment efficacy in PD-L1-positive EGFR-mutated NSCLC: the impact of antiangiogenic agents

**DOI:** 10.3389/fphar.2024.1391972

**Published:** 2024-08-05

**Authors:** Xuanhong Jin, Yang Pan, Cheng Cheng, Hangchen Shen, Chongya Zhai, Kailai Yin, Xinyu Zhu, Hongming Pan, Liangkun You

**Affiliations:** ^1^ Department of Medical Oncology, Sir Run Run Shaw Hospital, College of Medicine, Zhejiang University, Hangzhou, China; ^2^ Postgraduate Training Base Alliance of Wenzhou Medical University (Zhejiang Cancer Hospital), Hangzhou, China; ^3^ Department of Clinical Laboratory, Sir Run Run Shaw Hospital, College of Medicine, Zhejiang University, Hangzhou, China

**Keywords:** NSCLC, EGFR, TKIs, PD-L1, antiangiogenic therapy, immunotherapy

## Abstract

**Background:**

In individuals receiving treatment with epidermal growth factor receptor-tyrosine kinase inhibitors (EGFR-TKIs), those exhibiting positive PD-L1 expression might experience reduced progression-free survival (PFS). However, the effects on overall survival (OS) and the determination of efficacious treatment approaches are still not well-defined.

**Methods:**

In our retrospective study, we examined data from 193 NSCLC patients with advanced *EGFR* mutations who received first-line TKI treatments, treated at two centers of Shaw Hospital in Zhejiang, China. This analysis covered a period from 1 January 2016 to 30 April 2023.

**Results:**

Patients with PD-L1 positivity exhibited a markedly shorter average PFS (9.5 months versus 17.8 months, *P* < 0.001) and OS (44.4 months versus 65.7 months, *P* = 0.016) relative to those without PD-L1 expression. This difference in both PFS and OS remained statistically significant even after adjusting for multiple factors (*P* < 0.001 for PFS and *P* = 0.028 for OS). In the PD-L1-positive cohort, introducing combination antiangiogenic significantly extended both PFS (from 9.1 to 25.7 months, *P* = 0.026) and OS (from 42 to 53.5 months, *P* = 0.03). Post-first-line TKI therapy, 39.3% of PD-L1-positive patients and 54.5% of PD-L1-negative patients developed the T790M mutation (*P* = 0.212), with no notable difference in PFS from second-line TKI treatments between the groups. Additionally, subsequent combination therapy with immunotherapy markedly prolonged OS in the PD-L1-positive group. However, for PD-L1-negative patients, neither combination antiangiogenic therapy nor later-line immunotherapy demonstrated significant benefits in PFS or OS.

**Conclusion:**

For PD-L1-positive patients, combined antiangiogenic treatments and immunotherapy can significantly improve survival outcomes. In contrast, PD-L1-negative patients show less benefit from these therapies, highlighting the greater efficacy of these treatments in PD-L1-positive individuals.

## Introduction

Mutations in the epidermal growth factor receptor (EGFR) are common in non-small cell lung cancer (NSCLC), accounting for approximately 50% of cases in Asians and 25% in Caucasians (Lynch et al., 2004). These mutations are particularly prevalent in East Asian lung adenocarcinoma patients, females, and non-smokers (Shi et al., 2014). The introduction of EGFR tyrosine kinase inhibitors (TKIs) has transformed the therapeutic approach for advanced NSCLC patients with *EGFR* mutations, establishing them as the primary treatment option and significantly enhancing survival (Rosell et al., 2012). Despite the development of third-generation TKIs targeting the T790M resistance mutation (Mok et al., 2017), early resistance continues to be a major issue for many patients. Current research is focused on understanding the causes of this resistance and finding effective ways to counter it.

In the precision medicine era, the use of immune checkpoint inhibitors (ICIs) has significantly altered the approach to treating NSCLC (Mok et al., 2019). However, in patients with *EGFR*-mutant NSCLC, PD-L1 expression is generally lower (Soo et al., 2018), which correlates with less effective responses to ICIs in this subset (Gainor et al., 2016). Previous studies have shown that PD-L1 positive and negative patients have distinct molecular landscapes, which may significantly impact patient treatment and prognosis (Pisapia et al., 2022). Recent meta-analyses indicate that patients with PD-L1 positivity tend to have shorter progression-free survival (PFS) when treated with first-line EGFR TKIs (Peng et al., 2021). This is further evidenced by various studies examining the relationship between PFS, PD-L1 expression, and the use of third-generation TKIs (Yoshimura et al., 2021; Hsu et al., 2022; Hamakawa et al., 2023). The link between PD-L1 expression and overall survival (OS) in *EGFR* mutation-positive patients remains unclear. Some research suggests that PD-L1 positivity could be indicative of a poorer prognosis in these patients (Lin et al., 2015; Liu et al., 2021). Particularly, data on the PD-L1 status and OS in patients treated with third-generation TKIs are still lacking. Moreover, there is also research that points to PD-L1 positivity being associated with a reduced occurrence of the T790M mutation following first-line TKI therapy (Yang et al., 2020; Inomata et al., 2022), which might affect the selection and effectiveness of subsequent second-line treatments.

In the JO25567 phase II study and the NEJ026 phase III study, first-line erlotinib in combination with bevacizumab significantly prolonged PFS but did not show a significant benefit in OS (Seto et al., 2014; Saito et al., 2019). In contrast, some retrospective studies have shown that first-line or further-line combination bevacizumab can improve OS in patients treated with first-line TKIs (Tsai et al., 2021; You et al., 2022). Additionally, combining small molecule antiangiogenic agents such as anlotinib and apatinib is also a feasible strategy after progression on first-line TKIs (Han et al., 2024). A Taiwanese study specifically showed an OS benefit for patients with *EGFR* mutations only in those with poor prognostic factors, when treated with combination antiangiogenic therapy and first-line TKIs (Chen et al., 2022). Therefore, combination antiangiogenic therapy may be a promising option for patients with PD-L1 positivity.

We conducted a retrospective study aiming to determine the effect of PD-L1 status on prognosis among different subgroups of patients with *EGFR* mutations who received first-line TKI therapy, particularly whether PD-L1 status influences OS. We also compared the PFS benefit of first-line TKI combined with antiangiogenic therapy versus first-line TKI alone. Additionally, we evaluated the potential effects of antiangiogenic therapy and further-line immunotherapy on the OS of patients with varying PD-L1 statuses. Our study aims to provide tailored treatment strategies for clinical practice in first-line TKI therapy based on different PD-L1 statuses.

## Methods

### Research design and participant selection

This retrospective study was conducted at two facilities of Sir Run Run Shaw Hospital at Zhejiang University (Qingchun Hospital and Xiasha Hospital). It was ethically approved by the Ethics Committee of Sir Run Run Shaw Hospital, affiliated with the Zhejiang University School of Medicine, under the reference number 2024-2002-01. This approval ensured compliance with the ethical guidelines stipulated in the Declaration of Helsinki. Due to the retrospective nature of the research, the requirement for informed consent was waived by the Ethics Committee of Sir Run Run Shaw Hospital.

This research involved 193 lung cancer patients, enrolled between 1 January 2016, and 30 April 2023. The criteria for inclusion were: 1) Histologically verified NSCLC, 2) Stage III or IV lung cancer classification as per the 8th edition of the AJCC staging system, 3) Detection of an *EGFR* mutation, either an EGFR exon 21 p.L858R substitution or an EGFR exon 19 deletion, 4) Initial treatment with TKIs, and 5) Use of at least one standard chemotherapy regimen after TKI resistance. Exclusion criteria encompassed patients with alternate mutations or those who had undergone systemic treatment before starting TKI therapy.

Data collection in this study covered various demographic and clinical factors, including age, sex, smoking status, Eastern Cooperative Oncology Group Performance Status (ECOG PS) scores, clinical stage, and initial presence of brain or bone metastases and treatment regimen. The study also meticulously documented *EGFR* mutation status, any subsequent resistance mutations, and PD-L1 expression levels.

Details regarding the treatment regimen were comprehensively recorded, focusing on the first-line therapy administered to participants. The study tracked PFS during and after the TKI treatment period, alongside OS post-TKI therapy. PFS was measured from the beginning of TKI treatment until disease progression or death, whereas OS was determined from the start of TKI therapy until death.

### Antiangiogenic therapy and immunotherapy

Patients receiving combination antiangiogenic therapy have been treated in the first-line or subsequent-line, whereas combination immunotherapy have been treated in the subsequent-line only. First-line treatment is defined as the first systemic therapy given after diagnosis. Subsequent-line treatment is defined as any subsequent anticancer therapy administered after disease progression. The use of antiangiogenic agents and immunotherapy in first- and subsequent-line treatments is detailed in the Supplementary Table S1. Antiangiogenic drugs included bevacizumab (15 mg/kg once every 3 weeks), anlotinib (12 mg or 10 mg in 3-week cycles), and apatinib (500 mg in 4-week cycles). Immunotherapy is administered with anti-PD-1 or PD-L1 antibodies, including sintilimab (200 mg every 3 weeks), nivolumab (3 mg/kg every 2 weeks), pembrolizumab (200 mg/kg every 3 weeks), durvalumab (1,500 mg every 3 weeks), toripalimab (3 mg/kg every 3 weeks), or atezolizumab (1,200 mg every 3 weeks).

### PD-L1 expression and *EGFR* mutation analysis

PD-L1 immunohistochemistry (IHC) expression in tumor cells was determined using either the Ventana SP263 monoclonal antibody kit or the Dako 22C3 pharmDx kit. PD-L1 expression was categorized as positive when it was equal to or exceeded 1%, and as negative when it was below this threshold. For identifying *EGFR* mutations, our facility’s pathology department, or a third-party genetic testing service accredited by the College of American Pathologists (CAP), conducts genetic alteration tests on tumor tissues or cells. These samples are typically obtained through biopsy, surgical resection, or the centrifugation of pleural effusion sediments. The testing methodologies employed include NGS (Illumina) and polymerase chain reaction (PCR) techniques.

### Statistical analyses methods

For assessing differences in clinical characteristics between PD-L1 subgroups, the chi-square test was utilized. PFS and OS were estimated using the Kaplan-Meier method for survival analysis. Subgroup and interaction tests were conducted using the “jstable” function in R software. The Cox proportional hazards model was employed to evaluate survival time differences in PFS and OS. If univariate regression analysis shows a significant difference (*P* < 0.05), those variables will be included in the multivariate regression analysis. Statistical significance was determined using two-tailed tests, with *P* < 0.05 as the threshold for significance. Past statistical research indicates that in exploratory studies or when dealing with limited sample sizes, it is reasonable to use a looser significance level in interaction tests (Núñez et al., 2011). Therefore, in our interaction tests, a significance level of *P* < 0.1 was considered meaningful, specifically for interaction effects.

## Results

### Patient characteristics

The clinical characteristics of the 193 patients included in this study are described in Table 1, the median follow-up of the patients was 27.3 (1.4–89.2) months, all patients had adenocarcinomas, 121 (62.7%) patients were negative for PD-L1 status while 72 (37.3%) patients were positive for PD-L1 expression, 147 (76.1%) of them used 22C3 antibody test while 46 (23.9%) patients were tested with SP263 antibody, the median age of the patients was 64 (39–86) years, 103 (53.3%) were males and 90 (46.7%) were females, 147 (76.2%) patients had no history of smoking, 185 (96%) patients had an ECOG score between 0 and 1, 168 (87%) patients were stage IV while 25 (13%) patients were stage III, 46 (23.8%) and 85 (44%) patients were diagnosed with brain metastases and bone metastases at baseline, respectively. Genetic analysis revealed that 99 patients (51.3%) had the 19DEL mutation, whereas 94 patients (48.7%) exhibited the L858R mutation.

**TABLE 1:** Clinical characteristics for all patients.

Characteristics	PD-L1 (−) (N = 121)	PD-L1 (+) (N = 72)	*P*-value
Gender			
Female	56 (46.3%)	34 (47.2%)	1
Male	65 (53.7%)	38 (52.8%)	
Age			
<=65	72 (59.5%)	35 (48.6%)	0.186
>65	49 (40.5%)	37 (51.4%)	
Smoking history			
No	95 (78.5%)	52 (72.2%)	0.414
Yes	26 (21.5%)	20 (27.8%)	
ECOG			
0	49 (40.5%)	24 (33.3%)	0.604
1	67 (55.4%)	45 (62.5%)	
2	5 (4.1%)	3 (4.2%)	
Stage			
III	20 (16.5%)	5 (6.9%)	0.0899
IV	101 (83.5%)	67 (93.1%)	
Brain metastasis			
No	96 (79.3%)	51 (70.8%)	0.243
Yes	25 (20.7%)	21 (29.2%)	
Bone metastasis			
No	74 (61.2%)	34 (47.2%)	0.0826
Yes	47 (38.8%)	38 (52.8%)	
IHC			
22C3	95 (78.5%)	52 (72.2%)	0.414
SP263	26 (21.5%)	20 (27.8%)	
Mutation			
19DEL	67 (55.4%)	32 (44.4%)	0.187
L858R	54 (44.6%)	40 (55.6%)	
TKI Generation			
1	64 (52.9%)	29 (40.3%)	0.236
2	7 (5.8%)	5 (6.9%)	
3	50 (41.3%)	38 (52.8%)	

ECOG, eastern cooperative oncology group; TKI, tyrosine kinase inhibitor.

A total of 88 patients (45.6%) received a third-generation TKI as their first-line treatment. Antiangiogenic combination therapy was used in 91 patients (47.2%), with 31 of these (16.1%) receiving it as a first-line treatment. Among these, 10 patients experienced antiangiogenic combination therapy both in first-line and subsequent-line treatments. Immunotherapy was administered to 25 patients (13%), None received it as their initial treatment. Patients with PD-L1-positive and PD-L1-negative conditions essentially share broadly similar clinical features. PD-L1-positive patients had higher baseline tumor burden, such as higher baseline stage (*P* = 0.0899) and bone metastases (*P* = 0.0826).

### Correlation between PD-L1 status and patient outcomes

**FIGURE 1:**
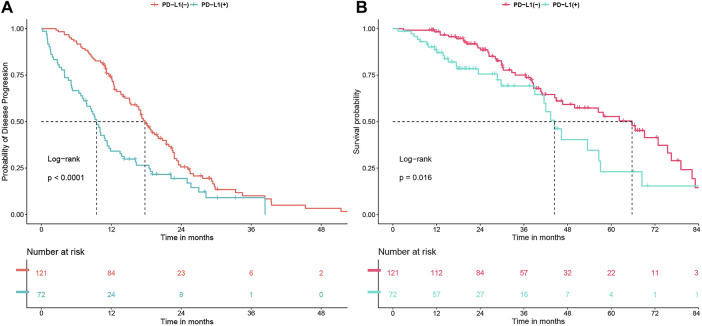
**(A)** Progression-free survival (PFS) and **(B)** Overall survival (OS) undergoing first-line tyrosine kinase inhibitor (TKI) treatment, analyzed in relation to PD-L1 status.

In the entire study group, the PFS was 15.1 months (ranging from 12.4 to 17.5 months), and the OS was 56.4 months (ranging from 44.4 to 68.4 months). Notably, patients with positive PD-L1 status demonstrated significantly reduced PFS and OS compared to those with negative PD-L1 status, with median PFS at 9.5 months versus 17.8 months (*P* < 0.0001) and median OS at 44.4 months versus 65.7 months (*P* = 0.016) as illustrated in Figure 1. Extensive univariate and multivariate Cox regression analyses revealed a consistent and significant link between PD-L1 status and both PFS (*P* < 0.001) and OS (*P* = 0.028), as shown in Table 2 and Table 3 respectively. Moreover, the addition of combination antiangiogenic therapy was identified as an independent variable impacting both PFS (*P* < 0.001) and OS (*P* = 0.021). Additionally, the presence of brain metastasis at diagnosis was recognized as an independent factor affecting PFS with initial-line therapy (HR 1.76, *P* = 0.024), along with the use of third-generation TKIs (HR 0.59, *P* = 0.002) and the L858R mutation (HR 1.54, *P* = 0.009). In terms of OS, bone metastasis is an independent prognostic factor (HR 1.65, *P* = 0.039).

**TABLE 2:** Univariable and multivariable analysis for progression-free survival (PFS) in all patients.

	Univariate analysis		Multivariate analysis	
Characteristics	HR (95% CI)	*P*‐value	HR (95% CI)	*P*‐value
PD-L1				
(−)	Reference		Reference	
(+)	1.90 (1.37–2.63)	*P* < 0.001	1.95 (1.40–2.73)	*P* < 0.001
Gender				
Female	Reference		—	
Male	1.09 (0.80–1.49)	*P* = 0.587	—	
Age				
<=65	Reference		—	
>65	0.86 (0.63–1.18)	*P* = 0.339	—	
Smoking history				
No	Reference		—	
Yes	1.02 (0.70–1.47)	*P* = 0.933	—	
ECOG				
0	Reference		Reference	
1–2	1.50 (1.07–2.10)	*P* = 0.019	1.27 (0.90–1.78)	*P* = 0.178
Stage				
III	Reference		—	
IV	1.46 (0.91–2.34)	*P* = 0.115	—	
Brain				
No	Reference		Reference	
Yes	1.69 (1.18–2.43)	*P* = 0.005	1.76 (1.21–2.57)	*P* = 0.003
Bone				
No	Reference		—	
Yes	1.20 (0.87–1.65)	*P* = 0.261	—	
Mutation				
19DEL	Reference		Reference	
L858R	1.48 (1.08–2.03)	*P* = 0.015	1.54 (1.11–2.13)	*P* = 0.009
TKI Generation				
1	Reference		Reference	
2	0.82 (0.41–1.62)	*P* = 0.560	0.69 (0.34–1.39)	*P* = 0.298
3	0.70 (0.50–0.97)	*P* = 0.032	0.59 (0.42–0.83)	*P* = 0.002
1st Line antiangiogenic				
No	Reference		Reference	
Yes	0.48 (0.30–0.75)	*P* = 0.001	0.40 (0.25–0.63)	*P* < 0.001

ECOG, eastern cooperative oncology group; TKI, tyrosine kinase inhibitor.

**TABLE 3:** Univariable and multivariable analysis for overall survival (OS) in all patients.

	Univariate analysis		Multivariate analysis	
Characteristics	HR (95% CI)	*P*‐value	HR (95% CI)	*P*‐value
PD-L1				
(−)	Reference		Reference	
(+)	1.79 (1.11–2.91)	*P* = 0.018	1.74 (1.06–2.84)	*P* = 0.028
Gender				
Female	Reference		—	
Male	1.27 (0.80–2.03)	*P* = 0.309	—	
Age				
<=65	Reference		—	
>65	1.35 (0.84–2.15)	*P* = 0.213	—	
Smoking history				
No	Reference		—	
Yes	1.24 (0.72–2.15)	*P* = 0.434	—	
ECOG				
0	Reference		—	
1–2	1.11 (0.69–1.77)	*P* = 0.673	—	
Stage				
III	Reference		—	
IV	1.06 (0.56–2.02)	*P* = 0.849	—	
Brain				
No	Reference		—	
Yes	1.42 (0.81–2.50)	*P* = 0.221	—	
Bone				
No	Reference		Reference	
Yes	1.69 (1.06–2.71)	*P* = 0.028	1.65 (1.03–2.66)	*P* = 0.039
Mutation				
19DEL	Reference		Reference	
L858R	1.53 (0.96–2.43)	*P* = 0.073	1.44 (0.90–2.30)	*P* = 0.128
TKI Generation				
1–2	Reference		—	
3	1.14 (0.66–1.95)	*P* = 0.644	—	
Antiangiogenic				
No	Reference		Reference	
Yes	0.55 (0.33–0.89)	*P* = 0.015	0.56 (0.34–0.92)	*P* = 0.021
Immunotherapy				
No	Reference		—	
Yes	0.77 (0.39–1.50)	*P* = 0.436	—	

ECOG, eastern cooperative oncology group; TKI, tyrosine kinase inhibitor.

### Subgroup analyses

**FIGURE 2:**
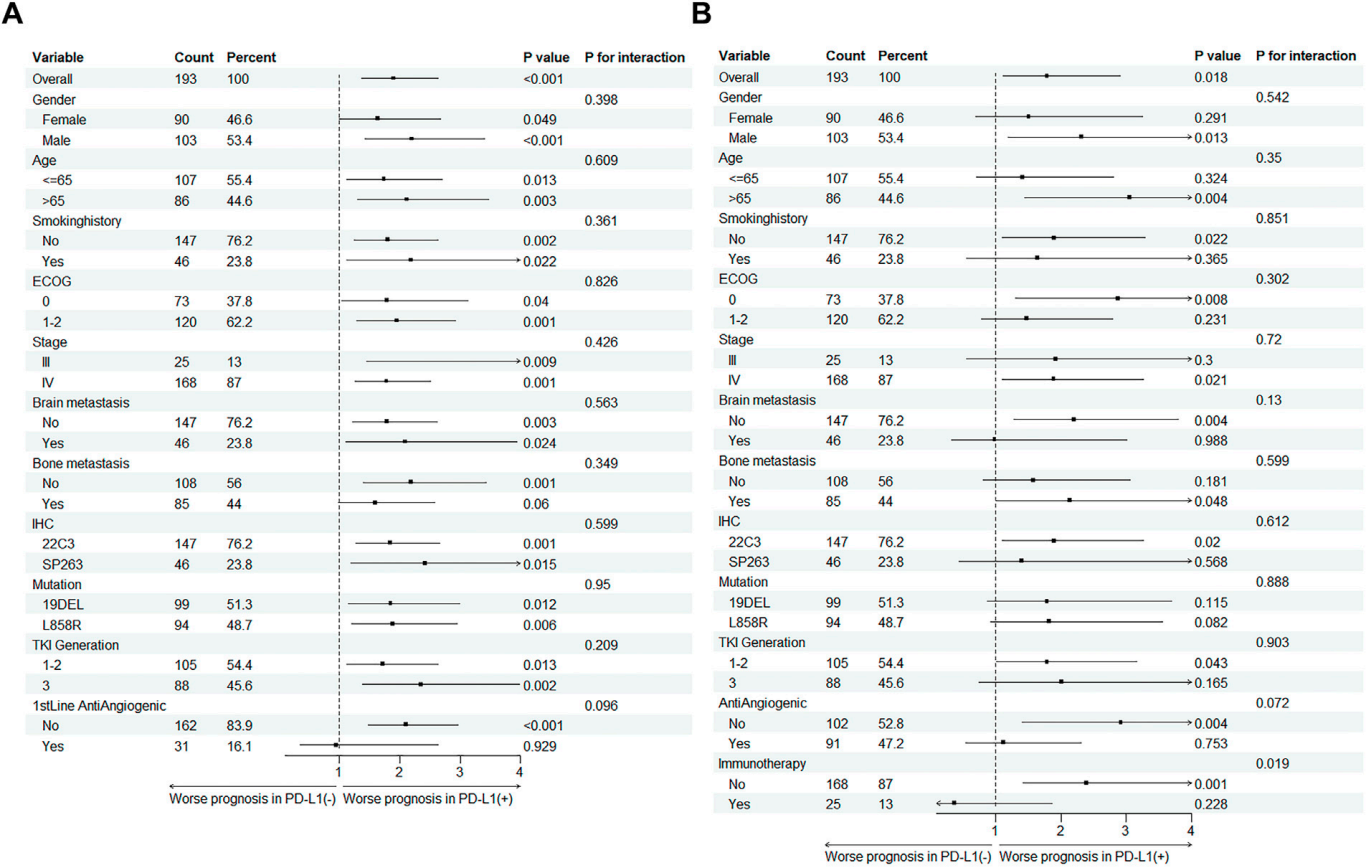
Subgroup analysis of PD-L1 status impact on **(A)** Progression-free survival (PFS) and **(B)** Overall survival (OS) across different subgroups in EGFR-Mutant lung cancer patients.

We subsequently conducted subgroup analyses focusing on PFS and overall survival OS as the endpoint events (Figure 2). For the PFS subgroup, almost all subgroups showed shorter PFS in PD-L1 (+) patients. However, no significant survival differences were detected between PD-L1 (+) and (−) groups within the cohort undergoing first-line antiangiogenic combination therapy (PD-L1 (+) vs. (−): HR 0.95, *P* = 0.929; *P* for interaction = 0.096). In the OS subgroup, we found no survival differences between PD-L1 (+) and (−) groups across various patient categories. Notably, this lack of difference was especially pronounced in those on antiangiogenic therapy (PD-L1 (+) vs. (−): HR 1.12, *P* = 0.753; P for interaction = 0.072), and patients receiving subsequent immunotherapy (PD-L1 (+) vs. (−): HR 0.37, *P* = 0.228; *P* for interaction = 0.019). In the treatment with third-generation TKIs, the PFS remained significantly lower in PD-L1 (+) patients (*P* = 0.002). Although no significant difference in OS was observed between third-generation TKI PD-L1 (+) and PD-L1 (−) patients (*P* = 0.165), a trend towards worse OS was noted for the PD-L1 (+) group (HR: 2.01, 95% CI 0.75–5.34).

### PD-L1 status-related survival stratified by antiangiogenic therapy

**FIGURE 3:**
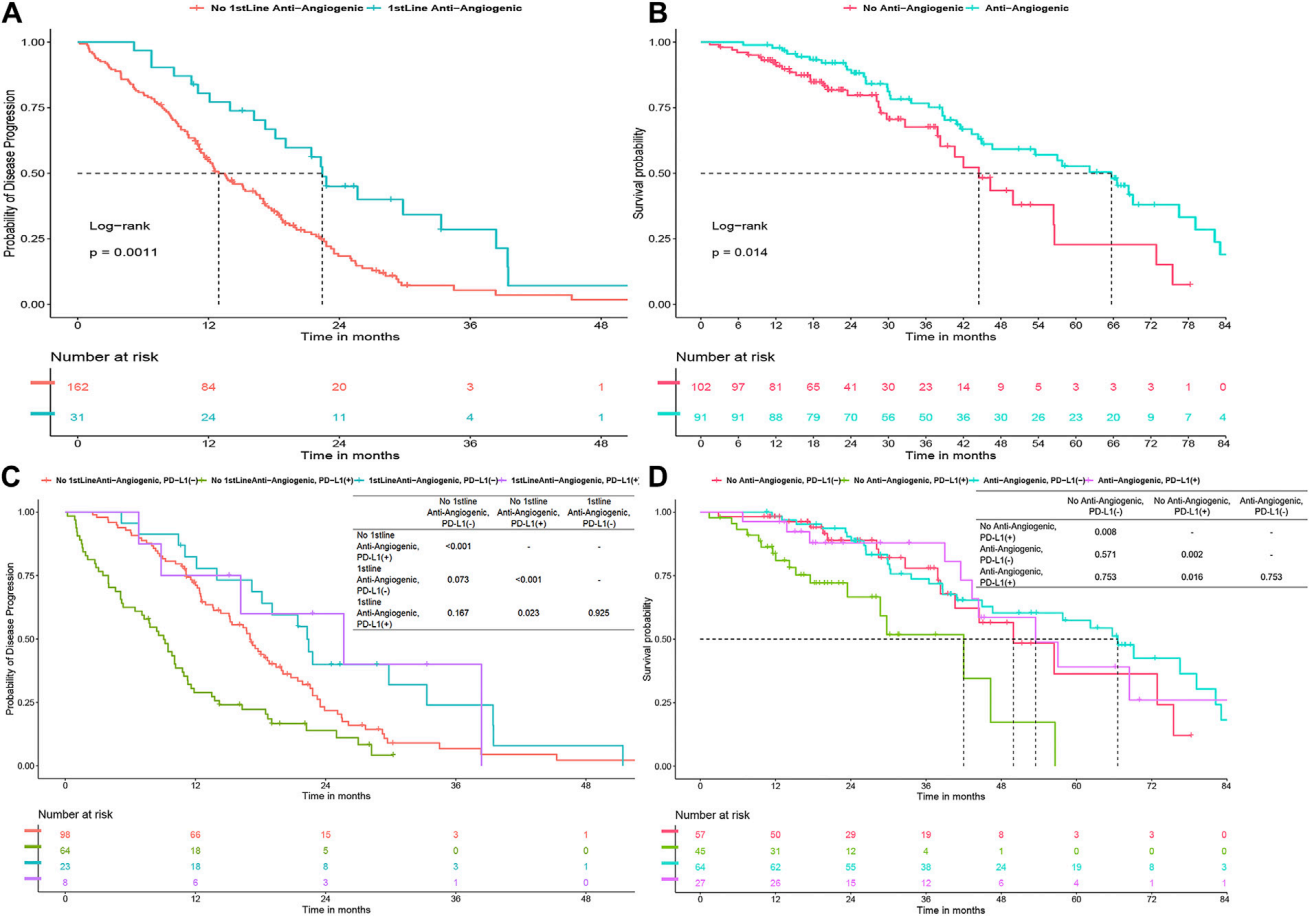
**(A)** Progression-free survival (PFS) for first-line TKI with/without anti-angiogenics. **(B)** Overall survival (OS) with anti-angiogenics across all treatments. **(C–D)** PFS and OS by PD-L1 status with anti-angiogenic use.

In the first-line treatment, the combination of antiangiogenic therapy with TKI therapy significantly improved PFS, extending it from 12.9 to 22.4 months (*P* = 0.001) (Figure 3A). This combination therapy also notably enhanced OS throughout the course of treatment, increasing it from 44.5 to 65.7 months (*P* = 0.014) (Figure 3B). When analyzing PD-L1 status within the patient group receiving antiangiogenic therapy, it was observed that this first-line combination therapy was particularly effective in improving PFS in PD-L1 (+) patients, raising it from 9.1 to 25.7 months (*P* = 0.026) (Figure 3C). In the same group, the combination markedly boosted OS from 42 to 53.5 months (*P* = 0.03) (Figure 3D), achieving a median survival time comparable to that of PD-L1 (−) patients. However, for PD-L1 (−) patients specifically, no significant improvement in OS and PFS was observed in antiangiogenic combination therapy group (*P* > 0.05).

### PD-L1 status concerning second-line TKI therapy and subsequent immunotherapy

**FIGURE 4:**
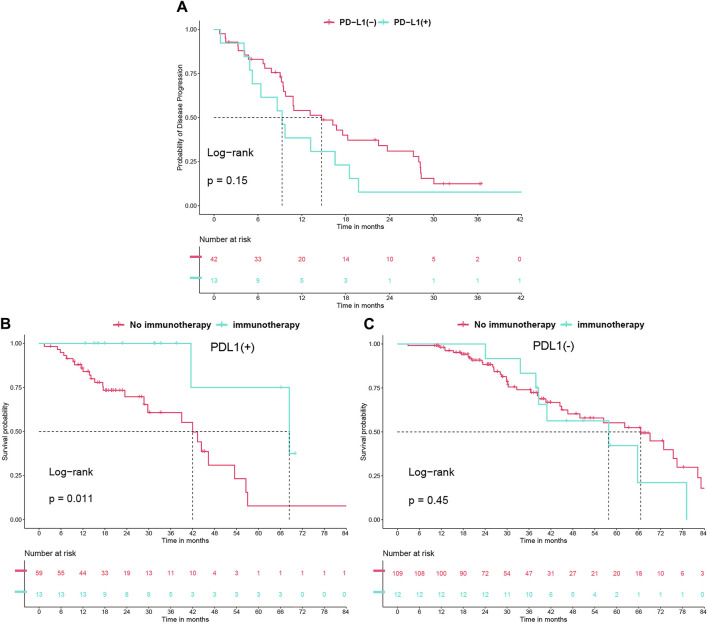
**(A)** Progression-free survival (PFS) undergoing second-line tyrosine kinase inhibitor (TKI) treatment, analyzed in relation to PD-L1 status. **(B)** Overall survival (OS) with immunotherapy use in PD-L1 (+) group. **(C)** OS with immunotherapy use in PD-L1 (−) group.

In our study, after excluding 31 non-progressing patients, 6 with initial T790M mutations, and 46 who didn’t test for the mutation post-resistance, 55 of the remaining 110 patients (50%) developed a T790M mutation following resistance to first-line TKI therapy. Of these, 13 of 33 PD-L1 (+) patients (39.3%) and 42 of 77 PD-L1 (−) patients (54.5%) (*P* = 0.212) had the mutation. These patients were then treated with second-line TKIs, switching to a third-generation TKI if initially treated with a first-generation TKI. Our analysis showed slightly worse but not statistically significant second-line PFS for PD-L1 (+) patients (9.3 vs. 14.7 months, *P* = 0.15) (Figure 4A). Additionally, immunotherapy significantly improved overall survival in PD-L1 (+) patients from 42 to 68.4 months (*P* = 0.011), but not in PD-L1 (−) patients (*P* = 0.45) (Figures 4B, C).

## Discussion

The patients harboring *EGFR*-sensitizing mutations are among those with the best prognosis in advanced NSCLC. However, some subgroups fare worse than others, such as patients who tested positive for both *EGFR* mutations and PD-L1 before initial therapy. In our retrospective analysis, we examined 193 patients with *EGFR*-mutated NSCLC receiving first-line TKI therapy across two centers at our institution. After a thorough evaluation of clinicopathological characteristics, we discovered that patients positive for PD-L1 exhibited a shorter PFS and OS compared to PD-L1-negative patients. These findings persisted even after adjustments for multiple factors.

The prognostic significance of PD-L1 expression in *EGFR*-mutated NSCLC patients, particularly regarding PFS with TKI, remains an area of ongoing research. Most studies (Su et al., 2018; Masuda et al., 2021; Yoshimura et al., 2021; Shiozawa et al., 2022; Hamakawa et al., 2023; Lei et al., 2023) and meta-analyses (Peng et al., 2021) have shown that patients with positive or high PD-L1 expression generally have poorer outcomes. This aligns with our findings, which bodes ill for this group of patients. Interestingly, we have discovered that those receiving first-line combined antiangiogenic therapy negated the impact of prognosis brought by PD-L1 expression, exhibited longer PFS in the PD-L1-positive subgroup.

Concerning OS, the impact of PD-L1 expression warrants further investigation. A meta-analysis indicated a marginally worse prognosis for patients with high PD-L1 expression (*P* = 0.070) (Peng et al., 2021). Given the proximity of this value to statistical significance and the limited number of studies, we conducted a more detailed analysis of OS across different PD-L1 statuses. Our results revealed a generally worse OS for PD-L1-positive patients overall. However, in subgroups such as patients with initial brain metastases, no significant statistical difference in OS was found. This suggests that the impact of PD-L1 status on OS might be limited. Moreover, we found that in patients who received combined antiangiogenic therapy and subsequent immunotherapy, PD-L1 status had almost no impact on OS. Therefore, although PD-L1 positivity may have a minor impact on prognosis, the combination of antiangiogenic therapy or subsequent immunotherapy effectively mitigated the OS loss in PD-L1 positive patients.

Previous research has primarily focused on the prognostic implications of PD-L1 expression in patients with *EGFR*-mutated NSCLC. However, there’s a significant gap in understanding how to improve outcomes for patients exhibiting high PD-L1 expression. Our study attempts to contribute to this area of research by exploring the potential efficacy of first-line TKI in combination therapy. We focused on the efficacy of combining first-line TKIs with antiangiogenic therapy, particularly in the context of PD-L1 expression. Our findings reveal that this combination therapy significantly enhances both PFS and OS in PD-L1-positive patients, effectively neutralizing the adverse prognostic effects typically associated with high PD-L1 expression. This improvement is likely due to the observed increase in VEGFA expression among PD-L1-positive lung adenocarcinomas (Koh et al., 2019), a phenomenon also noted in various other cancers (Shin et al., 2016; Koh et al., 2017; Fujii et al., 2020; Yang et al., 2021; Yu et al., 2023). VEGF can influence immune cells in various ways. One notable mechanism is through the enhancement of regulatory T cell (Treg) recruitment and activity, which can modulate immune responses. Furthermore, VEGF and other angiogenic factors such as angiopoietin-2 can impact the functionality of other immune cells, including dendritic cells and macrophages. VEGF can impair the antigen-presenting capacity of dendritic cells and modify their cytokine production, thereby influencing the overall immune response. Intriguingly, antiangiogenic therapy appears to counteract the pro-angiogenic factors stimulated by PD-L1, particularly through the STAT signaling pathway in NSCLC cell lines (Cavazzoni et al., 2024). To our knowledge, this is the first study to stratify the effect of antiangiogenic therapy by PD-L1 expression in *EGFR* mutant population, and therefore, first-line TKI in combination with antiangiogenic therapy could be a preferable option in the clinic for patients with *EGFR* mutations who are initially tested positive for PD-L1 or have high expression.

Some studies have suggested a correlation between PD-L1 status and the prevalence of T790M mutations (Yang et al., 2020; Inomata et al., 2022). Our research supports this association, finding that PD-L1-positive patients had fewer T790M mutations. This may give more credit to 3rd generation TKI in the first line setting. Additionally, we observed that PD-L1-positive patients treated with second-line TKIs had slightly worse prognoses, though the differences were not statistically significant. Larger-scale studies are needed to confirm the impact of PD-L1 expression on prognosis in second-line treatments.

Jinfei Si et al. reported that patients treated with immune checkpoint inhibitors (ICIs) in combination with antiangiogenic therapy experienced longer PFS and OS compared to those treated with ICIs and chemotherapy (Si et al., 2023). Yujing Li et al. found that subsequent immunotherapy significantly improved survival in *EGFR*-mutated patients with high PD-L1 expression after resistance to therapy (Li et al., 2023). In alignment with these findings, our analysis of subsequent ICI treatment according to different PD-L1 statuses revealed that PD-L1-positive patients benefited from immunotherapy even in the presence of *EGFR* mutation. This supports the use of immunotherapy in patients with high PD-L1 expression and *EGFR* mutation following the failure of first-line TKI treatment, a benefit not observed in PD-L1-negative patients.

In the field of PD-L1 expression and its prognostic relevance, the determination of a cut-off point remains a subject of debate. While a majority of previous studies have designated 50% as the threshold to differentiate between high and low PD-L1 expression (Hsu et al., 2022; Shiozawa et al., 2022), there is growing evidence of prognostic variances between PD-L1 positivity and negativity (Kobayashi et al., 2018; Hsu et al., 2019; Inomata et al., 2020; Lei et al., 2023). A recent study has proposed that a 20% cut-off point might more accurately reflect these prognostic differences (Hsu et al., 2022). This suggestion is particularly relevant in light of the substantial variability in PD-L1 expression detection caused by different antibodies and experimental conditions. Given that patients with *EGFR* mutations often exhibit very low PD-L1 expression (Schoenfeld et al., 2020), our study has chosen the 1% criterion. This decision aims to effectively address the heterogeneity issues arising from variations in PD-L1 detection methods, thereby improving the broad applicability and relevance of our findings in the context of diverse clinical scenarios.

One of the current issues is that patients with *EGFR* mutations are infrequently tested for PD-L1 expression due to the limited efficacy of immunotherapy in these cases. However, based on our findings, we recommend that patients with *EGFR* mutations undergo PD-L1 expression testing prior to initiating first-line TKI therapy. PD-L1 expression may have predictive value for prognosis and could predict the efficacy of combination anti-angiogenic therapy as well as subsequent immunotherapy.

This study, while offering valuable insights, is subject to certain limitations. Firstly, its retrospective design and relatively small sample size may introduce a degree of selection bias, albeit unintentionally. Secondly, to substantiate our findings more conclusively, we advocate prospective clinical trials designed to address the role of antiangiogenetic agents in patients with both *EGFR* mutations and PD-L1 expression. Such future research endeavors could provide more definitive evidence and further validate our conclusions.

## Data Availability

The datasets presented in this article are not readily available because due to confidentiality and ethical considerations, this data is not openly accessible. Requests to access the datasets should be directed to XJ, 2114894356@qq.com.
